# Network pharmacology and molecular docking-based investigations of Kochiae Fructus’s active phytomolecules, molecular targets, and pathways in treating COVID-19

**DOI:** 10.3389/fmicb.2022.972576

**Published:** 2022-08-05

**Authors:** Shakeel Ahmad Khan, Terence Kin Wah Lee

**Affiliations:** ^1^Department of Applied Biology and Chemical Technology, The Hong Kong Polytechnic University, Kowloon, Hong Kong SAR, China; ^2^State Key Laboratory of Chemical Biology and Drug Discovery, The Hong Kong Polytechnic University, Kowloon, Hong Kong SAR, China

**Keywords:** Kochiae Fructus, COVID-19, network pharmacology, molecular docking, molecular pathways

## Abstract

COVID-19 disease is caused by SARS-CoV-2. Hyper-inflammation mediated by proinflammatory cytokines is humans’ primary etiology of SARS-CoV-2 infection. Kochiae Fructus is widely used in China as traditional Chinese medicine (TCM) to treat inflammatory diseases. Due to its anti-inflammatory properties, we hypothesized that Kochiae Fructus would be a promising therapeutic agent for COVID-19. The active phytomolecules, targets, and molecular pathways of Kochiae Fructus in treating COVID-19 have not been explored yet. Network pharmacology analysis was performed to determine the active phytomolecules, molecular targets, and pathways of Kochiae Fructus. The phytomolecules in Kochiae Fructus were retrieved from the Traditional Chinese Medicine Systems Pharmacology (TCMSP) database, and their potential targets were predicted with the SwissTargetPrediction webserver. COVID-19-related targets were recovered from the GeneCards database. Intersecting targets were determined with the VENNY tool. The Protein-protein interaction (PPI) and Molecular Complex Detection (MCODE) network analyses were constructed using the Cytoscape software. Using the DAVID tool, gene ontology (GO) and Kyoto Encyclopedia of Genes and Genomes (KEGG) pathway enrichment analysis were performed on the intersecting targets. AutoDock Vina (version 1.2.0.) was used for molecular docking analysis. Six active phytomolecules and 165 their potential targets, 1,745 COVID-19-related targets, and 34 intersecting targets were identified. Network analysis determined 13 anti-COVID-19 core targets and three key active phytomolecules (Oleanolic acid, 9E,12Z-octadecadienoic acid, and 11,14-eicosadienoic acid). Three key pathways (pathways in cancer, the TNF signaling pathway, and lipid and atherosclerosis) and the top six anti-COVID-19 core targets (IL-6, PPARG, MAPK3, PTGS2, ICAM1, and MAPK1) were determined to be involved in the treatment of COVID-19 with active phytomolecules of Kochiae Fructus. Molecular docking analysis revealed that three key active phytomolecules of Kochiae Fructus had a regulatory effect on the identified anti-COVID-19 core targets. Hence, these findings offer a foundation for developing anti-COVID-19 drugs based on phytomolecules of Kochiae Fructus.

## Introduction

The severe acute respiratory syndrome coronavirus 2 (SARS-CoV-2) initially emerged in December 2019 in Wuhan, China, and then spread fast across the country, causing extensive respiratory infections (Rizwan et al., 2020). Till now, we have remained unsuccessful in halting the transmission of SARS-CoV-2 and its subsequent disease, “Corona Virus Disease 2019” (COVID-19). They are posing a continuous threat to human health and are claiming the deaths of numerous people over the globe daily (Tang et al., 2022). One of the most significant impediments in treating COVID-19 is the production of proinflammatory cytokines throughout the disease’s progression, referred to as cytokine release syndrome (CRS). CRS has been identified as humans’ primary etiology of SARS- and MERS-CoV infection (Channappanavar and Perlman, 2017; Mehta et al., 2020; Niu et al., 2021). COVID-19 patients have been observed to have systemic hyper-inflammation, also known as macrophage activation syndrome or cytokine storm (McGonagle et al., 2020). Reports demonstrate that a biomarker for CRS is an increase in pro-inflammatory cytokines such as IL-6. In patients with COVID-19, IL-6-induced CRS has been observed (Fehr et al., 2017; Ruan et al., 2020a; Zhang X. et al., 2020). Studies have reported that Tocilizumab, an IL-6 receptor antagonist, can be effective in suppressing cytokine storms in patients with COVID-19. However, early evidence from randomized clinical trials remains inconclusive (No author list, 2020; Niu et al., 2021). The only anti-COVID-19 therapies available are anti-SARS-CoV-2 vaccines such as BNT162b2, CoronaVac, ChAdOx1 nCoV-19, and mRNA-1273 which have been administered to people all over the globe (Tang et al., 2022). The vaccination program of anti-SARS-CoV-2 vaccines has successfully prevented thousands of deaths from COVID-19. Despite their therapeutic efficacy, these vaccines are reported to induce severe complications, including hypersensitivity myocarditis, Bell’s palsy, vaccine-induced immune thrombocytopenia, and thrombosis (VITT) (NICE, 2021; Kounis et al., 2022; Wan et al., 2022). As a corollary, developing more effective therapeutics with a reduced risk of adverse effects against COVID-19 remains a critical demand.

In this instance, traditional Chinese medicine (TCM) may be a viable alternative therapy for COVID-19. TCM has been used for thousands of years to treat a variety of diseases. Recent research has shown that TCM is quite beneficial in the treatment of COVID-19 (Liu et al., 2020; Ma Q. et al., 2020; Yang et al., 2020). The phytochemicals in TCM, such as polyphenols, flavonoids, alkaloids, and terpenoids, have been shown to effectively suppress pro-inflammatory cytokines (Zhou H. X. et al., 2020; El-Missiry et al., 2021; Alzaabi et al., 2022; Ge et al., 2022). Moreover, compared to conventional therapeutic drugs, the potential of TCM to treat just the symptoms of a disease while causing no damage to healthy cells makes it a more tempting choice for drug design and development.

Kochiae Fructus is the dried fruit of *Kochia scoparia* (L.) Schrad., has been used in TCM for over 2000 years as a topical and edible drug (Choi et al., 2014; Luo et al., 2021). Kochiae Fructus was initially reported in “Shennong Ben Cao Jing” as a “top grade” medicinal herb (Matsuda et al., 1999; Zou et al., 2021). Kochiae Fructus’s traditional efficacy was also reported in ancient medicinal books such as the Herbal Canon, Compendium of Materia Medica, etc., (Xiao et al., 2018; Zou et al., 2021). Kochiae Fructus has traditionally been used to treat back pain, malignant sores, skin itching, eczema, abnormal leucorrhea, male impotence, frequent urination, and eye conditions (Choi et al., 2014; Xiao et al., 2018; Luo et al., 2021; Zou et al., 2021). More than 150 phytomolecules have been identified in Kochiae Fructus that are characterized as heterocyclics, essential oils, organic acids, amino acids, carbohydrates (primarily mono- and disaccharides), flavonoids, and triterpenoids (Zou et al., 2021). Pharmacological studies demonstrated that extracts and components of Kochiae Fructus have anti-cancer, anti-microbial, anti-gastric mucosal damage, anti-edema, anti-nociceptive, anti-allergic, anti-inflammatory, anti-dermatitic, anti-pruritogenic, anti-itching, and hypoglycemic effects (Zou et al., 2021). Due to the presence of phytomolecules (polyphenols, flavonoids, terpenoids, etc.) and their anti-inflammatory properties, we hypothesized that Kochiae Fructus would be a promising therapeutic agent for COVID-19. The active molecules and mechanisms of action of Kochiae Fructus in treating COVID-19 have not been reported yet. Thus, we have provided several justifications for our hypothesis about the effectiveness of Kochiae Fructus in treating COVID-19.

**FIGURE 1:**
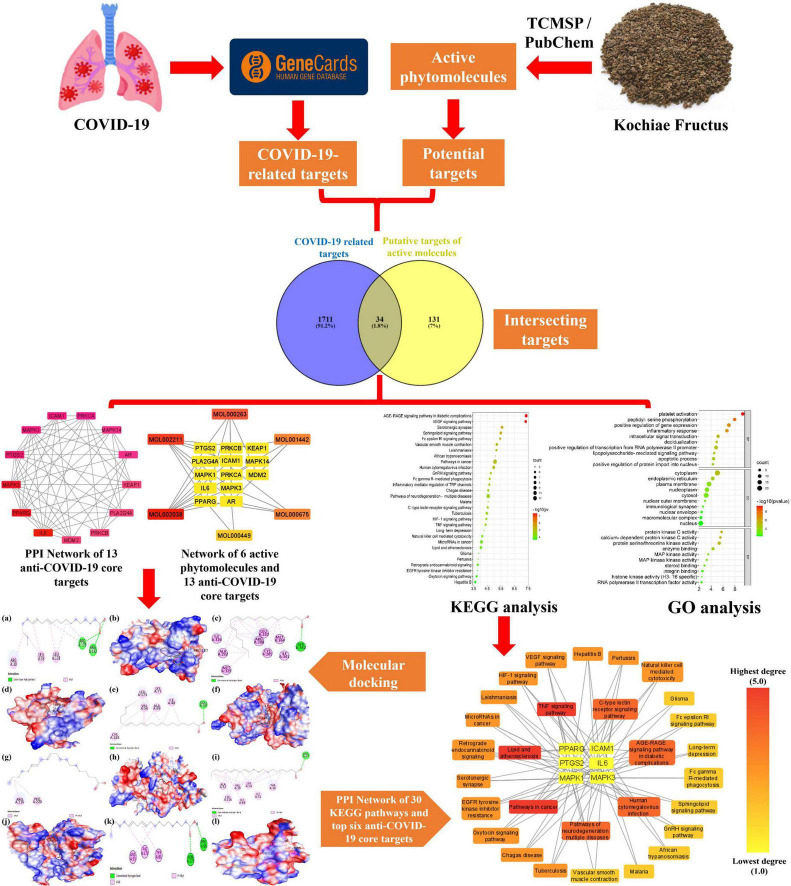
The workflow of the present study for predicting the active molecules and mechanism of action of Kochiae Fructus in treating COVID-19.

Network pharmacology and bioinformatics are emerging interdisciplinary fields involved in drug research and development. They utilize artificial intelligence as well as big data to identify active drug molecules and their molecular pathways (Xia, 2017). According to TCM’s holistic approach, network pharmacology intends to investigate the effectiveness of pharmaceuticals holistically, shifting research away from conventional one drug, one target mode, and toward a developing one drug, network targets paradigm (Zhou Z. et al., 2020). In the current study, we, therefore, employed different bioinformatics tools, including network pharmacology and molecular docking approaches, to predict biologically active molecules, molecular targets, as well as molecular pathways implicated in Kochiae Fructus’s anti-COVID-19 effects (Figure 1).

## Materials and methods

### Collection of Kochiae Fructus’s active molecules

We have retrieved the active phytomolecules of Kochiae Fructus utilizing the TCMSP (Version 2.3^1^) (Ru et al., 2014). The TCMSP database includes the relationships between each component’s absorption, distribution, metabolism, and excretion (ADME) characteristics. Oral bioavailability “OB” is a term that refers to “the rate and degree to which an active component or active moiety is absorbed from a therapeutic product that becomes accessible at the targeted site.” Drug Likeness “DL” is a qualitative paradigm for drug design that incorporates the ADME qualities of ingredients and established medications. Thus, the therapeutic phytomolecules of Kochiae Fructus that satisfied the requirements of both OB ≥ 20% and DL ≥ 0.10 were selected for further analysis (Chen et al., 2001; Ru et al., 2014; Ma X. et al., 2020; Wang et al., 2021).

### Screening of COVID-19-related targets

The COVID-19-related targets were retrieved from the GeneCards^®^ : The Human Gene Database^2^ by searching the keywords “Novel Coronavirus Pneumonia” and “Novel Coronavirus,” (Rebhan et al., 1997). After combining the targets of both keywords, the duplication of targets was removed using Venny 2.1 online database^3^ (Oliveros, 2007).

### SwissTargetPrediction of Kochiae Fructus’s active phytomolecules

By using the SwissTargetPrediction database,^4^ the potential targets of Kochiae Fructus’s active phytomolecules were acquired (Daina et al., 2019). The only potential targets with a probability score greater than 0 were selected for further analysis.

### Identification of intersecting targets

The intersection targets between the potential targets of the Kochiae Fructus’s active phytomolecules and the COVID-19-related targets were identified using the Venny 2.1 online database (see text footnote 3) (Oliveros, 2007). These identified intersecting targets were recognized as potential anti-COVID-19 key targets.

### Protein-protein interaction analysis

The potential anti-COVID-19 key targets (identified in section “Identification of intersecting targets”) were then assessed for protein-protein interaction (PPI) analysis using the STRING database (version 11.5, accessed on April 8, 2022^5^) at a confidence score > 0.4 and a species limited to “Homo sapiens” (von Mering et al., 2003; Chu et al., 2020). The string PPI analysis results were then uploaded to Cytoscape software (version 3.9.0, Boston, MA, the United States, accessed on April 8, 2022) to identify the potential anti-COVID-19 core targets (Lopes et al., 2010).

### Molecular Complex Detection analysis

The important modules in the PPI network of 34 potential anti-COVID-19 key targets were then determined by employing the Molecular Complex Detection (MCODE) plug-in in Cytoscape software (accessed on April 8, 2022) (Lopes et al., 2010). The conditions for MCODE analysis were Find clusters: in the whole network, Degree cutoff: 2, Node score cutoff: 0.2, K-core: 2, and Maximum Depth: 100 (Cao et al., 2021).

### Network construction between potential anti-COVID-19 (key and core) targets and Kochiae Fructus’s active phytomolecules

The network between anti-COVID-19 (key and core) targets and Kochiae Fructus’s active phytomolecules was further constructed by integrating them using Cytoscape software (version 3.9.0, Boston, MA, the United States, accessed on April 8, 2022) (Lopes et al., 2010; Chu et al., 2020).

### Enrichment analysis

Further enrichment analysis of GO functional and Kyoto Encyclopedia of Genes and Genomes (KEGG) pathway was performed on 34 potential anti-COVID-19 key targets (identified in section “Identification of intersecting targets”) using DAVID (Version 6.8)^6^ (Huang et al., 2008; Zhang et al., 2021). The GO terms were categorized into three types: cellular component (CC), biological process (BP), and molecular function (MF). By uploading the data to the Bioinformatics platform,^7^ the top 10 GO analysis data (BP, CC, and MF) and top 30 KEGG pathways were further exhibited in the form of an enrichment dot bubble (Weishengxin, 2022). The classical hypergeometric test was used to determine statistical significance. The adjusted *p*-value < 0.05 was utilized as the significant threshold in our investigation after utilizing the Benjamini–Hochberg method to control the false discovery rate (FDR) for multiple hypothesis testing (Chu et al., 2020).

### Molecular docking

Briefly, two-dimensional (2D) structures of Kochiae Fructus’s key active phytomolecules were obtained from the NCBI PubChem^8^ online database in Spatial Data File (SDF) format and three-dimensional (3D) structures were designed utilizing BIOVIA Discovery Studio Visualizer 2021 and saved in PDB file format (BIOVIA DS, 2016; NIH, 2022). Protein crystal structures of top-six potential anti-COVID-19 core targets were obtained in PDB format from the Protein Data Bank^9^ (Berman et al., 2000; RCSB PDB, 2022). The ligands and water molecules from the protein crystal structure complexes were retrieved using BIOVIA Discovery Studio Visualizer 2021 software. Following that, a grid of each protein was constructed using it, polar hydrogen was added, and the resulting protein was saved in PDB file format (BIOVIA DS, 2016). The PDB-formatted proteins were then uploaded to AutoDock Vina (version 1.2.0), and the Kollman and Gasteiger partial charges were applied. The key active phytomolecules of Kochiae Fructus were then transferred to AutoDock Vina in PDB format. Proteins and key active phytomolecules were converted to pdbqt format, and AutoDock Vina scripts were written for molecular docking (Trott and Olson, 2010). Finally, the acquired docked complexes were visualized using the software BIOVIA Discovery Studio Visualizer 2021 to assess the binding capacity of the key active phytomolecules and potential anti-COVID-19 core targets (BIOVIA DS, 2016). Binding energy less than zero shows that a ligand may spontaneously bind to the receptor. It is generally accepted that the lower the energy score of the ligand-receptor binding configuration, the greater the likelihood of binding (Trott and Olson, 2010; Chu et al., 2020).

## Results

### Active compounds screening

From the TCMSP database, 19 phytomolecules from Kochiae Fructus were retrieved. In addition, screening for active phytomolecules based on OB ≥ 20% and DL ≥ 0.10 resulted in identifying six active phytomolecules in Kochiae Fructus. Table 1 lists each of Kochiae Fructus’ six active phytomolecules.

**TABLE 1:** List of Kochiae Fructus’s active phytomolecules based on OB ≥ 20% and DL ≥ 0.10.

Phytomolecules ID	Phytomolecules name	Phytomolecules structure	Molecular weight	OB (%)	DL
MOL001442	Phytol	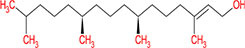	296.6	33.82	0.13
MOL002038	9E,12Z-octadecadienoic acid	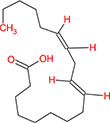	280.5	41.9	0.14
MOL002211	11,14-eicosadienoic acid	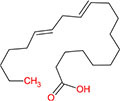	308.56	39.99	0.2
MOL000263	Oleanolic acid	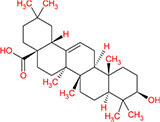	456.78	29.02	0.76
MOL000449	Stigmasterol	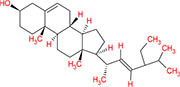	412.77	43.83	0.76
MOL000675	Oleic acid	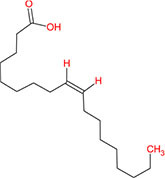	282.52	33.13	0.14

### Potential targets of Kochiae Fructus’s active phytomolecules

Using the SwissTargetPrediction online database, the potential targets of Kochiae Fructus’ active phytomolecules were identified. With a probability score > 0, 459 potential targets of six active phytomolecules were identified. 165 potential targets were chosen for further investigation after redundancies were eliminated.

### COVID-19-related targets

By searching the GeneCards database with the terms “Novel Coronavirus Pneumonia” and “Novel Coronavirus,” 1745 COVID-19-related targets were identified.

### Intersecting targets analysis

**FIGURE 2:**
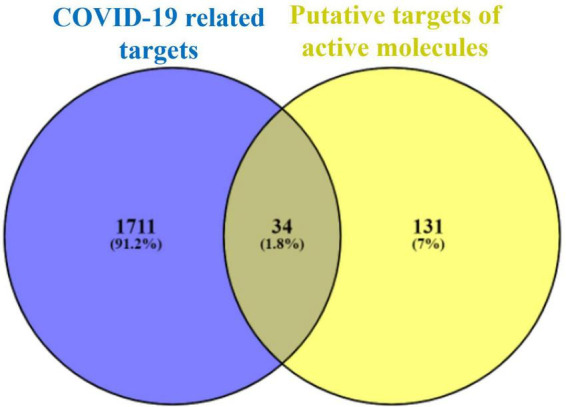
Intersecting targets between COVID-19-related targets and potential targets of Kochiae Fructus’ active phytomolecules.

Using the VENNY 2.1.0 online tool, the intersecting targets between the potential targets of Kochiae Fructus’ active phytomolecules and the COVID-19-related targets were analyzed, and 34 intersecting targets were determined as potential anti-COVID-19 key targets (Figure 2).

### Protein-protein interaction network construction and analysis

**FIGURE 3:**
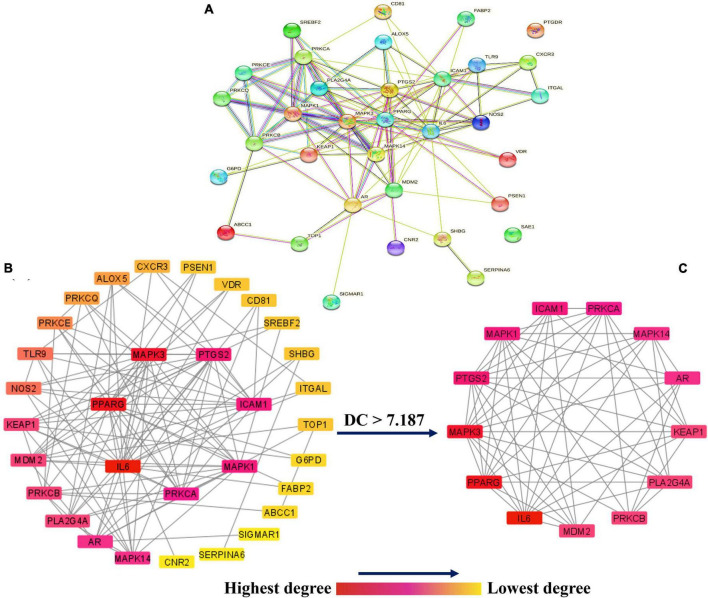
**(A)** The PPI network of 34 potential anti-COVID-19 key targets constructed by employing STRING. The PPI network of **(B)** 34 potential anti-COVID-19 key targets, and **(C)** 13 potential anti-COVID-19 core targets constructed by using the Cytoscape software. The color of each node changes from red (highest) to yellow (lowest) as its degree decreases. DC stands for degree centrality.

The PPI network was established by uploading 34 potential anti-COVID-19 key targets into the STRING database version 11.5, as illustrated in Figure 3A. The result reveals that the network has 34 nodes and 115 edges. Correspondingly, the average node degree, local clustering coefficient, expected number of edges, and PPI enrichment *p*-values were 6.76, 0.667, 32, and *p* < 0.0000001.

**FIGURE 4:**
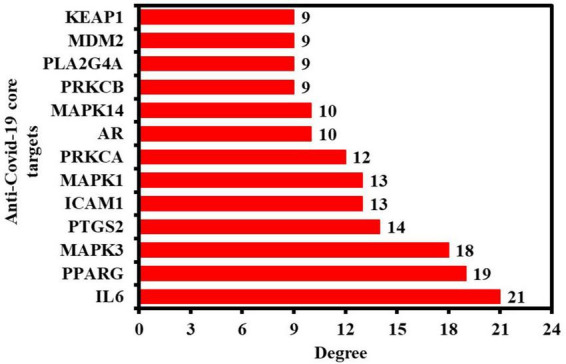
The 13 anti-COVID-19 core targets ranked by DC > average value of (7.187).

In addition, the PPI findings in a simple text data format (.tsv) file were then imported into the Cytoscape 3.9.0 software to visualize and analyze the PPI network. As seen in Figure 3B, the PPI network consisted of 32 nodes (after removing two unconnected nodes) and 115 edges, with an average shortest path length of 2.022 between all node pairings. The network’s density, diameter, and radius were 0.232, 4, and 3, respectively. The average number of neighbors, clustering coefficient, network heterogeneity, and network centralization were 7.188, 0.61, 0.75, and 0.475, respectively. As a PPI network node’s degree lowers, its color changes from red to yellow. The 13 nodes satisfying the degree centrality (DC) > average value (7.187) requirement were retrieved and identified as anti-COVID-19 core targets (Figure 3C). The 13 anti-COVID-19 core targets sorted by DC are shown as a bar graph in Figure 4. The top six anti-COVID-19 core targets, IL-6, PPARG, MAPK3, PTGS2, ICAM1, and MAPK1, were selected for molecular docking analyses with key active phytomolecules of Kochiae Fructus identified in section “Network of anti-COVID-19 targets and the Kochiae Fructus’s active phytomolecules.”

### Molecular Complex Detection analysis

**FIGURE 5:**
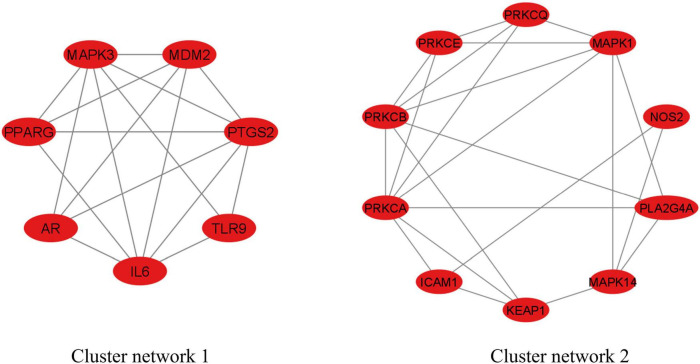
MCODE analysis of PPI network of 34 potential anti-COVID-19 key targets.

The MCODE analysis identified two cluster networks inside the PPI network of 34 anti-COVID-19 key targets, as seen in Figure 5. Cluster network 1 consisted of 7 nodes and 17 edges, with a score of 5.667. The components of cluster network 1 were IL6, AR, PPARG, MAPK3, MDM2, PTGS2, and TLR9. In cluster network 1, IL6, PPARG, MAPK3, and PTGS2 have extensive interconnections with multiple targets. Cluster network 2, on the other hand, has 10 nodes and 22 edges with a score of 4.889. PRKCA, PRKCB, PRKCCE, PRKCQ, MAPK1, NOS2, PLA2G4A, MAPK14, KEAP1, and ICAM1 were all part of Cluster Network 2. PRKCA, PRKCB, and MAPK1 are the highly interconnected targets in cluster network 2. Moreover, both cluster networks have found the top six anti-COVID-19 core targets. Cluster network 1 had four of them (IL6, PPARG, MAPK3, and PTGS2). Two of them (ICAM1 and MAPK1) were found in cluster network 2; thus, both the PPI network and MCODE analyses yielded consistent findings.

### Network of anti-COVID-19 targets and the Kochiae Fructus’s active phytomolecules

**FIGURE 6:**
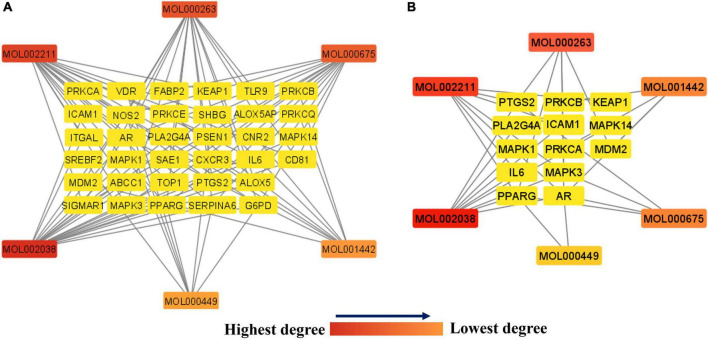
**(A)** Network of 34 potential anti-COVID-19 key targets and the Kochiae Fructus’s active phytomolecules. **(B)** The hub network of 13 potential anti-COVID-19 core targets and the Kochiae Fructus’s active phytomolecules. As the degree of each node decreases in both networks, the color changes from red (highest degree) to yellow (lowest degree).

As shown in Figure 6A, the network between the 34 potential anti-COVID-19 key targets and the active phytomolecules of Kochiae Fructus was established using Cytoscape software 3.9.0. It consists of 40 nodes and 86 edges. Each edge represents the interaction between active phytomolecules and potential anti-COVID-19 key targets. In addition, the network’s diameter and radius were, respectively, four and two. A node’s degree reflects the number of edges connecting it to other network nodes. As the degree of a node rises, its color changes from yellow (lowest degree) to red (highest degree).

**FIGURE 7:**
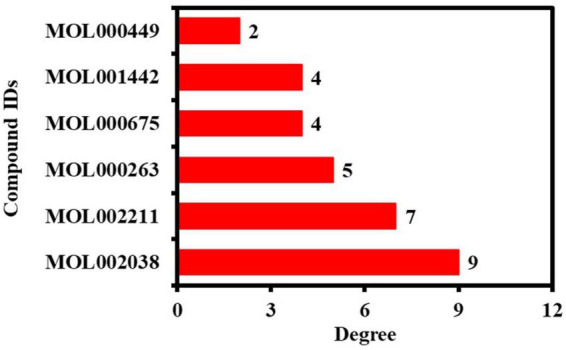
Kochiae Fructus’s key active phytomolecules in relation to hub network degree values.

In addition, the hub network between the 13 potential anti-COVID-19 core targets and the active phytomolecules of Kochiae Fructus was constructed using the Cytoscape software version 3.9.0, as shown in Figure 6B. All six active phytomolecules of Kochiae Fructus have been shown to interact with thirteen potential anti-COVID-19 core targets. The six active phytomolecules of Kochiae Fructus with their degree values in the hub network are presented as a bar graph in Figure 7. MOL002038 (9E,12Z-octadecadienoic acid), MOL002211 (11,14-eicosadienoic acid), and MOL000263 (oleanolic acid) are, respectively, the top three active phytomolecules that interact with nine, seven, and five anti-COVID-19 core targets (DC ≥ 5). Furthermore, the network findings revealed that single key active phytomolecules may interact with multiple anti-COVID-19 core targets and that multiple key active phytomolecules can interact synergistically with a single anti-COVID-19 core target. Thus, our results verified the combinatorial interaction mechanism between numerous anti-COVID-19 core targets and multiple key active phytomolecules in Kochiae Fructus.

### Gene ontology enrichment analysis

**FIGURE 8:**
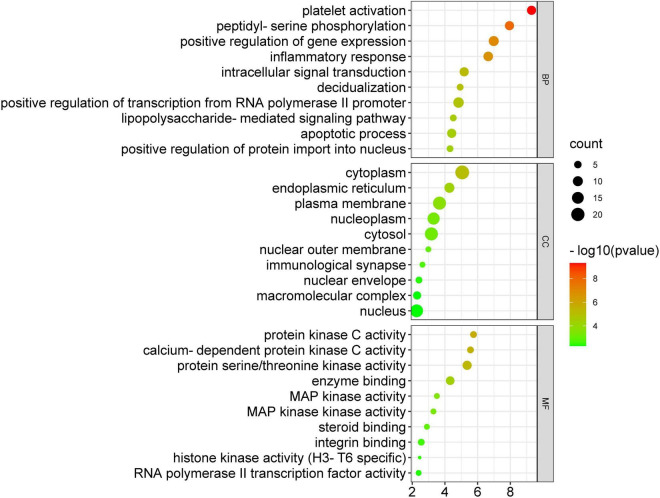
GO enrichment analysis of 34 potential anti-COVID-19 key targets.

The 34 potential anti-COVID-19 key targets were subjected to enrichment analysis of GO terms. Figure 8 depicts the top 10 enrichment terms of MF, CC, and BP for 34 potential anti-COVID-19 key targets of Kochiae Fructus. According to GO enrichment analysis, the gene targets associated with BP are embroiled in platelet activation, peptidyl-serine phosphorylation, inflammatory response, positive regulation of gene expression, intracellular transduction, positive regulation of transcription from RNA, polymerase II promoter, apoptotic process, etc. Gene targets in CC are primarily found in the cytoplasm, plasma membrane, nucleoplasm, cytosol, nucleus, endoplasmic reticulum, etc. GO enrichment analysis indicates further that protein serine/threonine kinase activity, calcium-dependent protein kinase activity, protein kinase C activity, enzyme binding, integrin binding, MAPK kinase activity, etc., dominate the enriched MF ontology.

### Kyoto Encyclopedia of Genes and Genomes analysis

**FIGURE 9:**
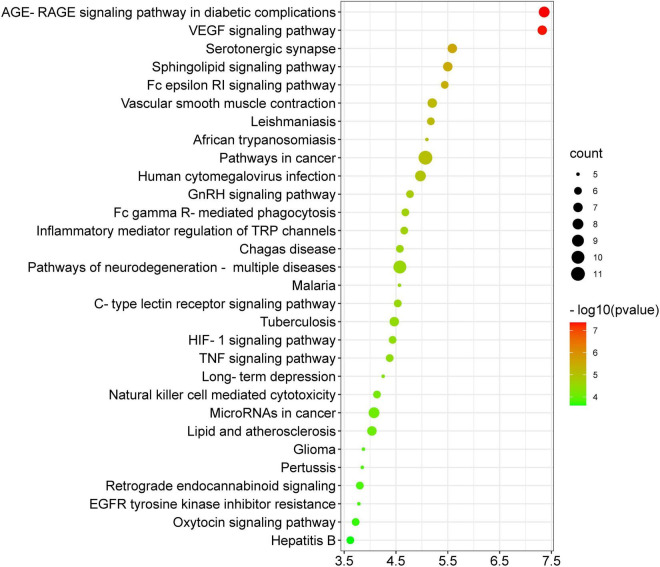
The top thirty enriched KEGG pathways of 34 potential anti-COVID-19 key targets.

Figure 9 depicts the thirty most significant KEGG pathways of the thirty-four potential anti-COVID-19 key targets. The KEGG pathway enrichment study suggested that Kochiae Fructus anti-COVID-19 key targets may be implicated in the AGE-RAGE signaling pathway in diabetic complications, the VEGF signaling pathway, cancer pathways, neurodegeneration-multiple diseases pathways, inflammatory mediator regulation of TRP channels, GnRH signaling pathway, Fc epsilon RI signaling pathway, Sphingolipid signaling pathway, TNF signaling pathway, HIF-1 signaling pathway, etc. These pathways may contribute substantially to the underlying mechanisms of Kochiae Fructus in alleviating COVID-19.

### Network between top six anti-COVID-19 core targets and thirty enriched KEGG pathways

**FIGURE 10:**
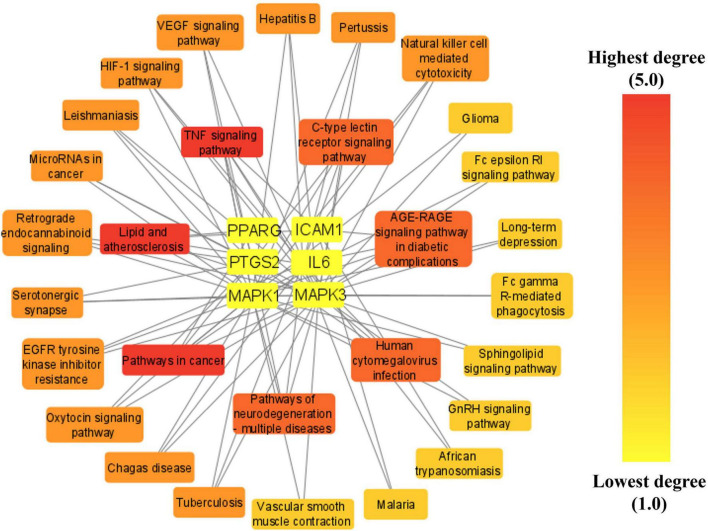
Network between top six anti-COVID-19 core targets and thirty enriched KEGG.

A network was established connecting the top six anti-COVID-19 core targets and their related pathways to determine the key pathways involved in the anti-COVID-19 effects of Kochiae Fructus’ active phytomolecules. The network findings revealed that five anti-COVID-19 core targets followed the pathways in cancer (IL6, MAPK1, PPARG, PTGS2, and MAPK3) (degree = 5), TNF signaling pathway (IL6, MAPK1, PTGS2, ICAM1, and MAPK3) (degree = 5), and Lipid and atherosclerosis (IL6, MAPK1, PPARG, ICAM1, and MAPK3) (degree = 5) (Figure 10).

**FIGURE 11:**
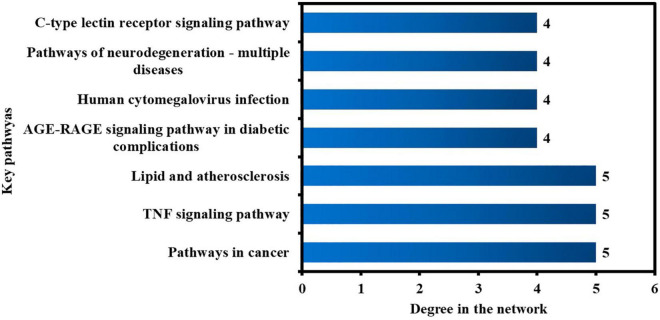
Seven key pathways in the network ranked by DC > average value (3.03).

Furthermore, the pathways in the network were sorted by DC > average value (3.03) to identify the key pathways. As illustrated in Figure 11, seven significant pathways were determined and shown in a bar graph with their degree values in the network. By regulating the expression of anti-COVID-19 core targets, these seven pathways may contribute considerably to the anti-COVID-19 effects of the active phytomolecules in Kochiae Fructus.

### Molecular docking studies

**FIGURE 12:**
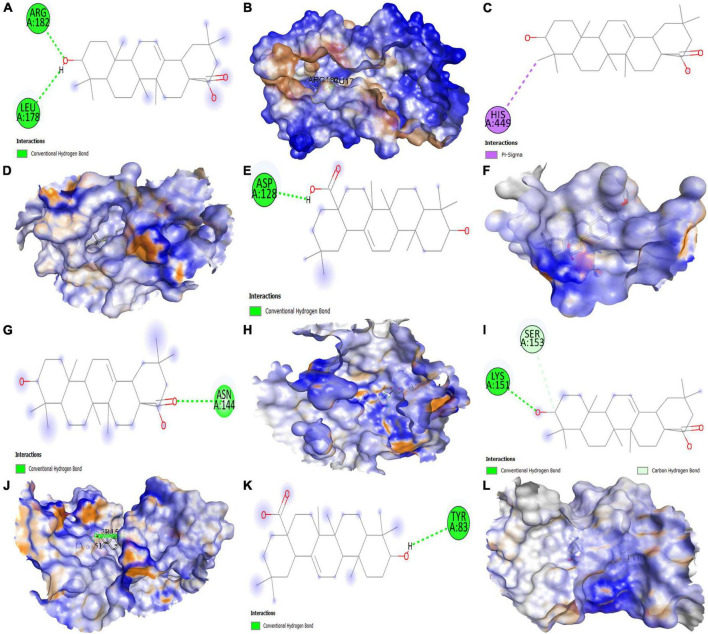
Molecular docking results. Binding of oleanolic acid (MOL000263) with **(A,B)** (2D and 3D) IL6, **(C,D)** (2D and 3D) PPARG, **(E,F)** (2D and 3D) MAPK3, **(G,H)** (2D and 3D) PTGS2, **(I,J)** (2D and 3D) MAPK1, and **(K,L)** (2D and 3D) ICAM1, respectively.

**FIGURE 13:**
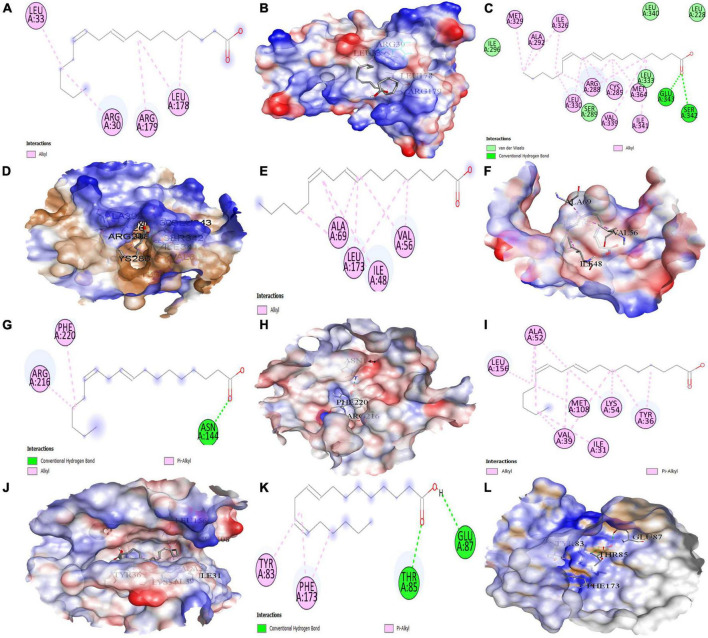
Molecular docking results. Binding of 9E,12Z-octadecadienoic acid (MOL002038) with **(A,B)** (2D and 3D) IL6, **(C,D)** (2D and 3D) PPARG, **(E,F)** (2D and 3D) MAPK3, **(G,H)** (2D and 3D) PTGS2, **(I,J)** (2D and 3D) MAPK1, and **(K,L)** (2D and 3D) ICAM1, respectively.

The top six anti-COVID-19 core targets, IL-6, PPARG, MAPK3, PTGS2, ICAM1, and MAPK1, were molecularly docked with the three key active phytomolecules of Kochiae Fructus. Table 2 displays the outcomes of molecular docking in terms of docking scores. Figures 12A–L, 13A–L and Supplementary Figures 1A–L present in 2D and 3D the docked complexes of key active phytomolecules and anti-COVID-19 core targets with the lowest docking scores. The docking score represents an active phytomolecules (ligands) affinity for anti-COVID-19 core targets (receptor). The lower the docking score, the better the ligand’s interaction with the receptor (Trott and Olson, 2010; Khan and Lee, 2022). Docking scores < –4.25, < –5.0, and < –7.0, respectively, correspond to the existence, good, and strong docking activity between the key active phytomolecules and the anti-COVID-19 core targets (Xie et al., 2021).

**TABLE 2:** Presents the binding affinities of Kochiae Fructus’s key active phytomolecules with the top six anti-COVID-19 core targets.

Phytomolecule IDs	Phytomolecule names	Binding affinities (kcal/mol)					
		IL6	PPARG	MAPK3	PTGS2	MAPK1	ICAM1
MOL000263	Oleanolic acid	–6.1	–1.0	–8.0	–4.9	–9.3	–5.9
MOL002038	9E,12Z-octadecadienoic acid	–4.5	–6.1	–5.5	–2.9	–5.9	–4.7
MOL002211	11,14-eicosadienoic acid	–4.1	–6.0	–5.6	–3.1	–6.3	–4.8

The results demonstrate that the MOL000263 (Oleanolic acid) exhibited a strong binding affinity with IL-6 by presenting a docking score of –6.1 (Table 2) and interacted with amino acid residues (ARG182 and LEU178) through conventional hydrogen bonding interaction (Figure 12A). MOL002038 (9E,12Z-octadecadienoic acid) and MOL002211 (11,14-eicosadienoic acid) demonstrated strong binding affinity with PPARG by exhibiting docking scores (–6.1 and –6.0) respectively, compared to MOL000263 (oleanolic acid), which presented a –1.0-docking score and interaction with amino acid residue (HIS449) *via* pi-sigma bond interaction (Figure 12C). The docking activity of all three key active phytomolecules with MAPK3 ranged from strong to excellent. MOL000263 (Oleanolic acid) provided the best docking score (–8.0) and was coupled with amino acid residue (ASP128) through conventional hydrogen bonding interaction (Figure 12E). Compared to the other two key active phytomolecules, MOL000263 (Oleanolic acid) coupled with PTGS2’s amino acid residues (ASN144) through conventional hydrogen bond interaction and exhibited a docking score of –4.9 (Figure 12G). All three active phytomolecules displayed a high affinity for binding with MAPK1. MOL000263 (Oleanolic acid) coupled with MAPK1’s amino acid residues (LYS151 and SER153) through conventional hydrogen bonding and carbon-hydrogen bonding, respectively, to achieve the best docking score (–8.0) (Figure 12I). The docking scores of MOL002038 (9E,12Z-octadecadienoic acid), MOL002211 (11,14-eicosadienoic acid), and MOL000263 (Oleanolic acid) with ICAM1 were –4.7, –4.8, and –5.9, respectively. These results suggested that the key active phytomolecules of Kochiae Fructus seemed to have a regulatory impact on the anti-COVID-19 core targets investigated, including IL-6, PPARG, MAPK3, PTGS2, ICAM1, and MAPK1. In addition, the findings of molecular docking and network pharmacology screening were consistent, indicating the validity of network pharmacology in this research.

## Discussion

This work investigated Kochiae Fructus’s active phytomolecules and molecular pathways for COVID-19 therapy. Based on OB ≥ 20 % and DL ≥ 0.10, six compounds were identified as active phytomolecules in Kochiae Fructus (Table 1). MOL002038 (9E,12Z-octadecadienoic acid), MOL002211 (11,14-eicosadienoic acid), and MOL000263 (oleanolic acid) are the top three key active phytomolecules interacting with more than four anti-COVID-19 core targets. In addition, the network analysis demonstrated that a single key active phytomolecule may interact with many anti-COVID-19 core targets and that several key active phytomolecules can interact synergistically with a single anti-COVID-19 core target. Consequently, our findings revealed a combinatorial interaction between the numerous anti-COVID-19 core targets and multiple key active phytomolecules in Kochiae Fructus for COVID-19 therapy.

The PPI network analysis reveals that multiple targets, including IL-6, PPARG, MAPK3, PTGS2, ICAM1, MAPK1, etc., are involved in the anti-COVID-19 effects of Kochiae Fructus’ active phytomolecules (Figures 3, 4). In COVID-19 patients, IL6 was markedly overexpressed and attributed to inflammation. CRS mediated by IL-6 is prevalent among COVID-19 patients and is accountable for their severe acute respiratory distress. By suppressing IL6 with tocilizumab, Xu et al. showed a reduction in CRS and a quick improvement in symptoms in patients (Ruan et al., 2020b; Wang et al., 2020; Xu et al., 2020). Reports indicate that overactivation/upregulation of MAPK (MAK1 and MAPK3) mediates the production of inflammatory cytokines such as IL-1β, IL6, IL10, TNF-alpha, IL4, and INF-gamma (de Souza et al., 2014; Grimes and Grimes, 2020; Oh et al., 2021) and these cytokines induce CRS. Furthermore, MAPK overactivation has been linked to thrombotic events and vascular endothelial infections in critically sick COVID-19 patients (Grimes and Grimes, 2020; Varga et al., 2020; Zhang Y. et al., 2020; Zhou F. et al., 2020). Furthermore, owing to MAPK overactivation, alveolar tissues are injured, resulting in reduced ventilation, acute lung injury, and acute respiratory distress syndrome (Goel et al., 2021). Consequently, targeting IL6, MAPK1, and MAPK3 may be a viable alternative therapeutic approach for treating CRS and COVID-19. On the other hand, allergic responses are caused by soluble ICAM1 in nasal epithelial cells. ICAM1 is found in a significant proportion of bronchial asthma patients. ICAM1 offers a suitable atmosphere for the coronavirus to infiltrate and live inside the human nose (Winther et al., 2002; Sharma et al., 2021). Nagashima et al. (2020) reported that COVID-19 endothelial cells overexpressed ICAM1 relative to controls, which might attract leukocytes (endotheliitis) and send intracellular signals leading to a pro-inflammatory state. A proinflammatory condition causes systemic endothelial dysfunction and endothelial cell death. The adhesion molecules’ chronic inflammatory signaling contributes to thrombosis (Nagashima et al., 2020). PTGS2 (COX2) is involved in controlling the homeostasis of the organism. Inflammatory stimuli modulate PTGS2 expression (Ricciotti and Fitzgerald, 2011). Chen et al. reported that SARS-CoV-2 infection increased the expression of PTGS2 in animal systems and human cell cultures (Chen et al., 2021). Inhibiting ICAM1 and PTGS2 with inhibitors can be a potential therapeutic strategy in treating COVID-19. PPARs, especially PPARG, is responsible for regulating glucose and lipid metabolism. Additionally, they control homeostasis and inhibit the production of certain proinflammatory cytokines. However, alterations in their expression (downregulation) result in the inflammation involved with COVID-19 etiology (Ciavarella et al., 2020). Desterke et al. (2020) reported the suppression of PPARG in severe COVID-19 and proposed that it contributes to monocyte/macrophage-mediated inflammatory storm. Thus, activating PPARG with an agonist might be a potential COVID-19 therapeutic modality.

According to GO enrichment analysis, Kochiae Fructus’ active phytomolecules might be implicated in affecting COVID-19 targets associated with multiple BP, including platelet activation, peptidyl-serine phosphorylation, inflammatory response, positive regulation of gene expression, intracellular transduction, positive regulation of transcription from RNA, polymerase II promoter, apoptotic process, etc. Platelet hyperactivation has been observed in COVID-19 patients. Platelets secrete procoagulants in critically ill individuals. They influence immune responses by interacting with other immune cells, resulting in severe thromboinflammation. Targeting these pathways may reduce immune response (Jevtic and Nazy, 2022; Uzun et al., 2022). Patients with COVID-19 have been shown to have elevated levels of peptidyl-serine phosphorylation (Ong et al., 2021; Chatterjee and Thakur, 2022). The inflammatory response is caused by an overactivation of the innate and adaptive immune systems and the invasion of SARS-CoV-2, which invades and activates numerous immune cells (Tan et al., 2021). Multiple intracellular transductions, such as TNF-alpha, mTOR, NF-κB, etc., are implicated in the pathogenesis of COVID-19 (Farahani et al., 2022). Kochiae Fructus’ active phytomolecules impacting COVID-19 targets associated with numerous CC are predominantly located in the cytoplasm, plasma membrane, nucleoplasm, cytosol, nucleus, endoplasmic reticulum, etc. Moreover, these COVID-19 targets perform multiple MF such as protein serine/threonine kinase activity, calcium-dependent protein kinase activity, protein kinase C activity, enzyme binding, integrin binding, MAPK kinase activity, etc.

The KEGG pathway enrichment investigation revealed that the molecular pathways driving the anti-COVID-19 effects of Kochiae Fructus’ active phytomolecules might be involved in the AGE-RAGE signaling pathway in diabetic complications, the VEGF signaling pathway, cancer pathways, neurodegeneration-multiple diseases pathways, inflammatory mediator regulation of TRP channels, GnRH signaling pathway, Fc epsilon RI signaling pathway, Sphingolipid signaling pathway, TNF signaling pathway, HIF-1 signaling pathway, etc. Moreover, the network analysis of the top six anti-COVID-19 core targets and thirty enriched KEGG pathways demonstrated that seven pathways, pathways in cancer, TNF signaling pathway, lipid and atherosclerosis, AGE-RAGE signaling pathway in diabetic complications, human cytomegalovirus infection, pathways of neurodegeneration-multiple diseases, and C-type lectin receptor signaling pathways, are the key pathways ranked by DC > average value (3.03). Among them, pathways in cancer and the TNF signaling pathway are the core pathways followed by five anti-COVID-19 core targets. The abnormal expression of targets (IL6, MAPK1, PPARG, PTGS2, and MAPK3) leads to cell proliferation and a variety of cancer types. Regulating pathways in cancer may modulate their expression. The pathophysiology of COVID-19 is also attributed to the expression/activation of these targets. In COVID-19 treatment, their expression may also be regulated by targeting cancer-related pathways. For instance, Hayashi et al. demonstrated that antineoplastic drugs inhibit MAPK and prevent SARS-CoV-2 replication (Hayashi and Konishi, 2021). Reports show that an overactive TNF signaling pathway has been identified as a key contributor to the development and severity of COVID-19. Its overactivation is induced by various factors (innate immune activation, cytokines release, high glucose level, etc.), leading to the onset of inflammation and the eventual failure of multiple organs (heart failure, insulin resistance, lung damage, blood clotting, etc.) (Ablamunits and Lepsy, 2022). Hence, these pathways may play a significant role in the underlying mechanisms of Kochiae Fructus’ active phytomolecules in COVID-19 alleviation.

## Conclusion

In this research, we have successfully determined the active phytomolecules and molecular pathways of Kochiae Fructus for COVID-19 therapy. Thirteen anti-COVID-19 core targets and three key active phytomolecules have been determined. This research also showed that, out of thirteen targets, the top six (IL-6, PPARG, MAPK3, PTGS2, ICAM1, and MAPK1) are likely to be implicated in the anti-COVID-19 effects of Kochiae Fructus’ active phytomolecules. The underlying mechanisms through which Kochiae Fructus’ active phytomolecules demonstrate anti-COVID-19 effects are the inhibition/regulation of the multiple BP (platelet activation, peptidyl-serine phosphorylation, inflammatory response, positive regulation of gene expression, intracellular transduction, positive regulation of transcription from RNA, polymerase II promoter, apoptotic process, etc.). We identified three key pathways (pathways in cancer, the TNF signaling pathway, and lipid and atherosclerosis) involved in the treatment of COVID-19 with active phytomolecules of Kochiae Fructus. Consequently, our results demonstrated a synergistic impact between the multiple anti-COVID-19 core targets, multiple molecular pathways, and key active phytomolecules in Kochiae Fructus for treating COVID-19. The findings of molecular docking revealed that the key active phytomolecules of Kochiae Fructus had a regulatory effect on the anti-COVID-19 core targets. Furthermore, molecular docking and network pharmacology screening results were consistent, demonstrating network pharmacology’s validity in this research. Hence, these findings offer a foundation for developing anti-COVID-19 drugs further in the future based on phytomolecules of Kochiae Fructus.

## Data availability statement

The raw data supporting the conclusions of this article will be made available by the authors, without undue reservation.

## Author contributions

SK and TL: conceptualization, validation, investigation, and writing—review and editing and original draft preparation. and editing. SK: methodology, software, formal analysis, data curation, and visualization. TL: supervision, project administration, and funding acquisition. Both authors have read and agreed to the published version of the manuscript.

## Footnotes

1 https://old.tcmsp-e.com/tcmsp.php, accessed on April 8, 2022

2 https://www.genecards.org/, accessed on April 8, 2022

3 https://bioinfogp.cnb.csic.es/tools/venny/, accessed on April 8, 2022

4 http://www.swisstargetprediction.ch/, accessed on April 8, 2022

5
https://string-db.org/

6 https://david.ncifrf.gov/, accessed on April 8, 2022

7 http://www.bioinformatics.com.cn/, accessed on April 8, 2022

8 https://pubchem.nlm.nih.gov/, accessed on April 9, 2022

9 https://www.rcsb.org/, accessed on April 9, 2022
