# Supplementary material for: Network pharmacology and molecular docking-based investigations of Kochiae Fructus’s active phytomolecules, molecular targets, and pathways in treating COVID-19
Source: Front Microbiol. 2022 Aug 5;13:972576. doi: 10.3389/fmicb.2022.972576 (PMC9389148; doi:10.3389/fmicb.2022.972576)
Supplement: Supplementary file 1 [file Image_1.pdf]

Supplementary Material

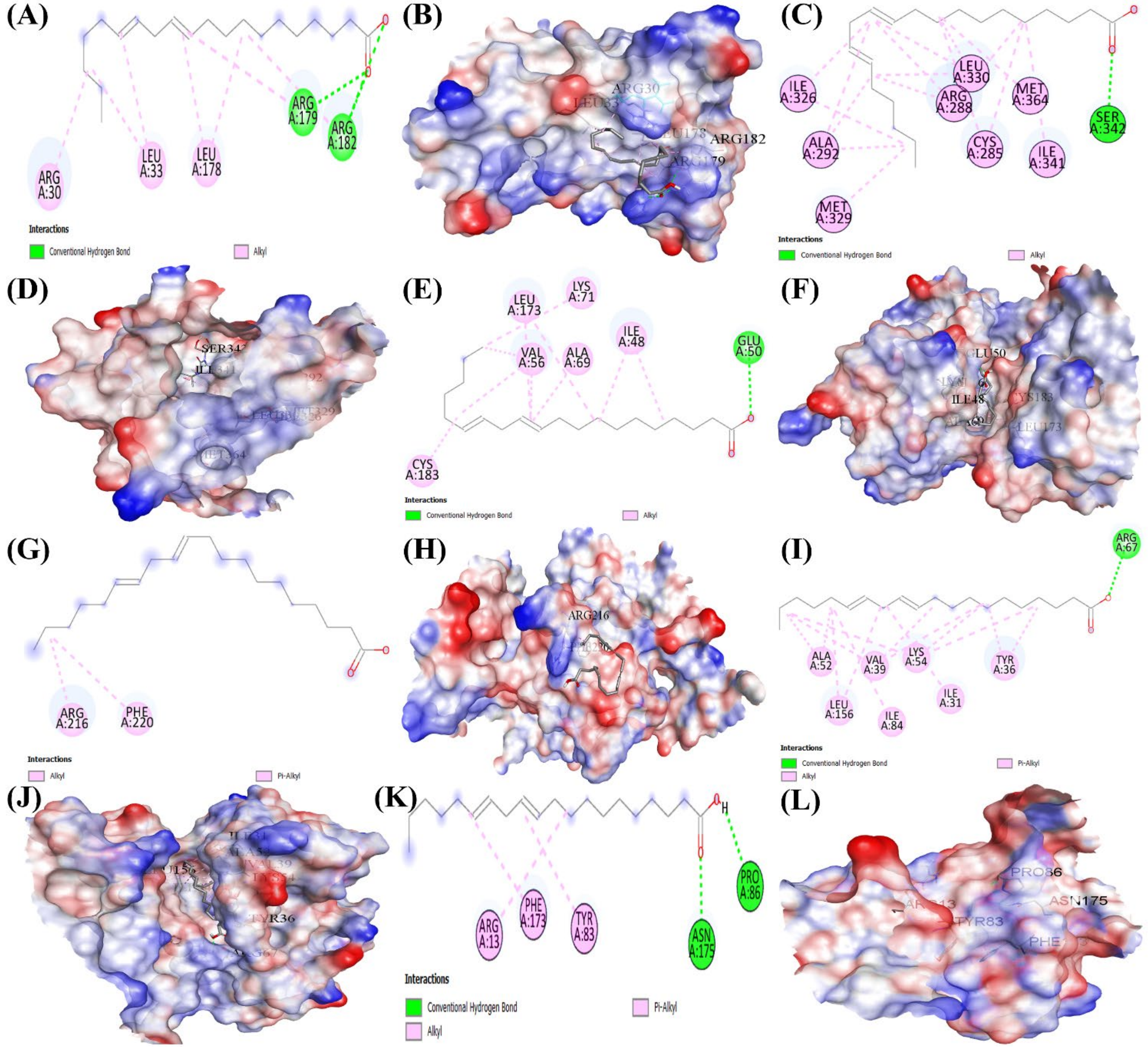

Figure S1. Molecular docking results. Binding of 11,14-eicosadienoic acid (MOL002211) with (**A** & **B**) (2D & 3D) IL6, (**C** & **D**) (2D & 3D) PPARG, (**E** & **F**) (2D & 3D) MAPK3, (**G** & **H**) (2D & 3D) PTGS2, (**I** & **J**) (2D & 3D) MAPK1, and (**k** & **L**) (2D & 3D) ICAM1, respectively.
